# Rapid imaging of thymoma and thymic carcinoma with a fluorogenic probe targeting γ-glutamyltranspeptidase

**DOI:** 10.1038/s41598-023-30753-2

**Published:** 2023-03-07

**Authors:** Daisuke Yoshida, Mako Kamiya, Shun Kawashima, Takafusa Yoshioka, Haruaki Hino, Atsuki Abe, Kyohhei Fujita, Ryosuke Kojima, Aya Shinozaki-Ushiku, Yasuteru Urano, Jun Nakajima

**Affiliations:** 1 Department of Thoracic Surgery, Graduate School of Medicine, The University of Tokyo, 7-3-1 Hongo, Bunkyo-ku, Tokyo, 113-8655 Japan; 2 Laboratory of Chemical Biology and Molecular Imaging, Graduate School of Medicine, The University of Tokyo, 7-3-1 Hongo, Bunkyo-ku, Tokyo, 113-0033 Japan; 3 Department of Thoracic Surgery, Kansai Medical University, 2-3-1 Shinmachi, Hirakata, Osaka 573-1191 Japan; 4 Department of Pathology, Graduate School of Medicine, The University of Tokyo, 7-3-1 Hongo, Bunkyo-ku, Tokyo, 113-8655 Japan; 5 Graduate School of Pharmaceutical Sciences, The University of Tokyo, 7-3-1 Hongo, Bunkyo-ku, Tokyo, 113-0033 Japan

**Keywords:** Molecular biology, Biomarkers

## Abstract

In recent years, thoracoscopic and robotic surgical procedures have increasingly replaced median sternotomy for thymoma and thymic carcinoma. In cases of partial thymectomy, the prognosis is greatly improved by ensuring a sufficient margin from the tumor, and therefore intraoperative fluorescent imaging of the tumor is especially valuable in thoracoscopic and robotic surgery, where tactile information is not available. γ-Glutamyl hydroxymethyl rhodamine green (gGlu-HMRG) has been applied for fluorescence imaging of some types of tumors in the resected tissues, and here we aimed to examine its validity for the imaging of thymoma and thymic carcinoma. 22 patients with thymoma or thymic carcinoma who underwent surgery between February 2013 and January 2021 were included in the study. Ex vivo imaging of specimens was performed, and the sensitivity and specificity of gGlu-HMRG were 77.3% and 100%, respectively. Immunohistochemistry (IHC) staining was performed to confirm expression of gGlu-HMRG's target enzyme, γ-glutamyltranspeptidase (GGT). IHC revealed high GGT expression in thymoma and thymic carcinoma in contrast to absent or low expression in normal thymic parenchyma and fat tissue. These results suggest the utility of gGlu-HMRG as a fluorescence probe for intraoperative visualization of thymomas and thymic carcinomas.

## Introduction

Thymoma and thymic carcinoma, although relatively rare^1^, are the most common mediastinal tumors in adults. Surgery for thymoma and thymic carcinoma has traditionally been performed by median sternotomy, but in recent years, thoracoscopic surgery and robotic surgery have become technically feasible^2^. Furthermore, simple thymomectomy involving resection of the tumor alone has increasingly been performed instead of extended thymectomy^3^. As tactile information is not available during thoracoscopic surgery and robotic surgery, the visual information provided by fluorescent probes can play a key role, especially in cases of simple thymomectomy, where it is important to leave an appropriate resection margin.

We have already reported the usefulness of fluorescence imaging using γ-glutamyl hydroxymethyl rhodamine green (gGlu-HMRG) for lung cancer, hepatocellular carcinoma, and breast cancer^4–6^. gGlu-HMRG itself is nonfluorescent but converted to highly fluorescent hydroxymethyl rhodamine green (HMRG) upon reaction with γ-glutamyltranspeptidase (GGT)^7^ (Supplementary Figs. S1 and S2). GGT is highly expressed in various types of cancer, so we anticipated that it might also be highly expressed in thymic epithelial tumors^4–6^. Therefore, in this study, we aimed to validate fluorescence imaging using gGlu-HMRG for thymic epithelial tumors.

## Results

### Ex vivo fluorescence imaging of specimens from thymoma and thymic carcinoma patients

**Figure 1:**
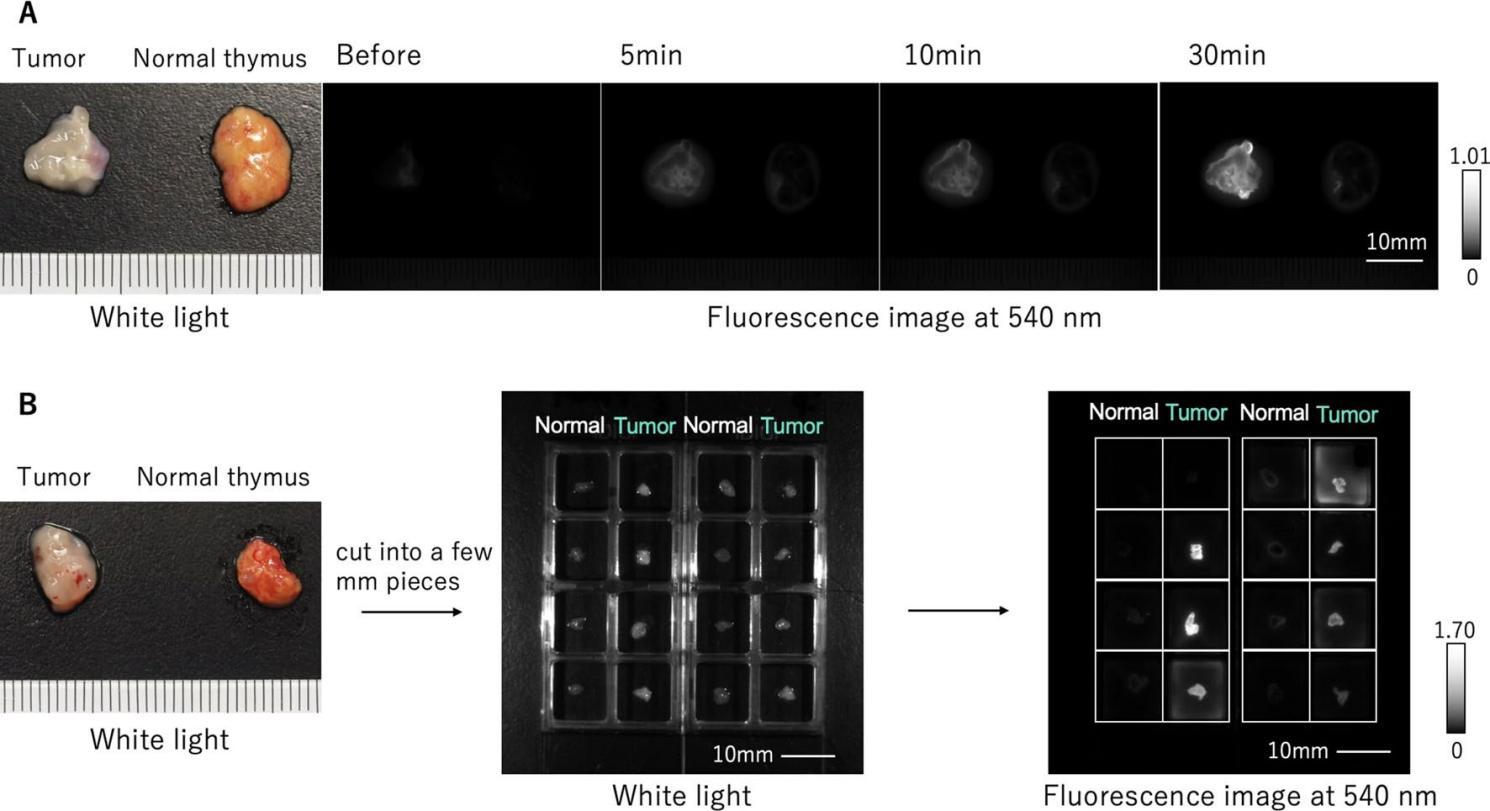
Ex vivo fluorescence imaging of specimens from thymoma and thymic carcinoma patients. A solution of 50 μM gGlu-HMRG was applied to tumor and normal tissue, and images were captured before and at 5, 10, and 30 min after application by Maestro with an appropriate exposure time. The fluorescence image at 540 nm was extracted. Probe solution was prepared with PBS (−) containing 0.5% v/v DMSO as a cosolvent. One graduation on the ruler is 1 mm. (**A**) Ex vivo fluorescence imaging of relatively large specimens of thymic tumor and normal tissues. (**B**) Ex vivo fluorescence imaging of cut pieces of thymic tumor and normal tissues a few millimeters in size.

**Figure 2:**
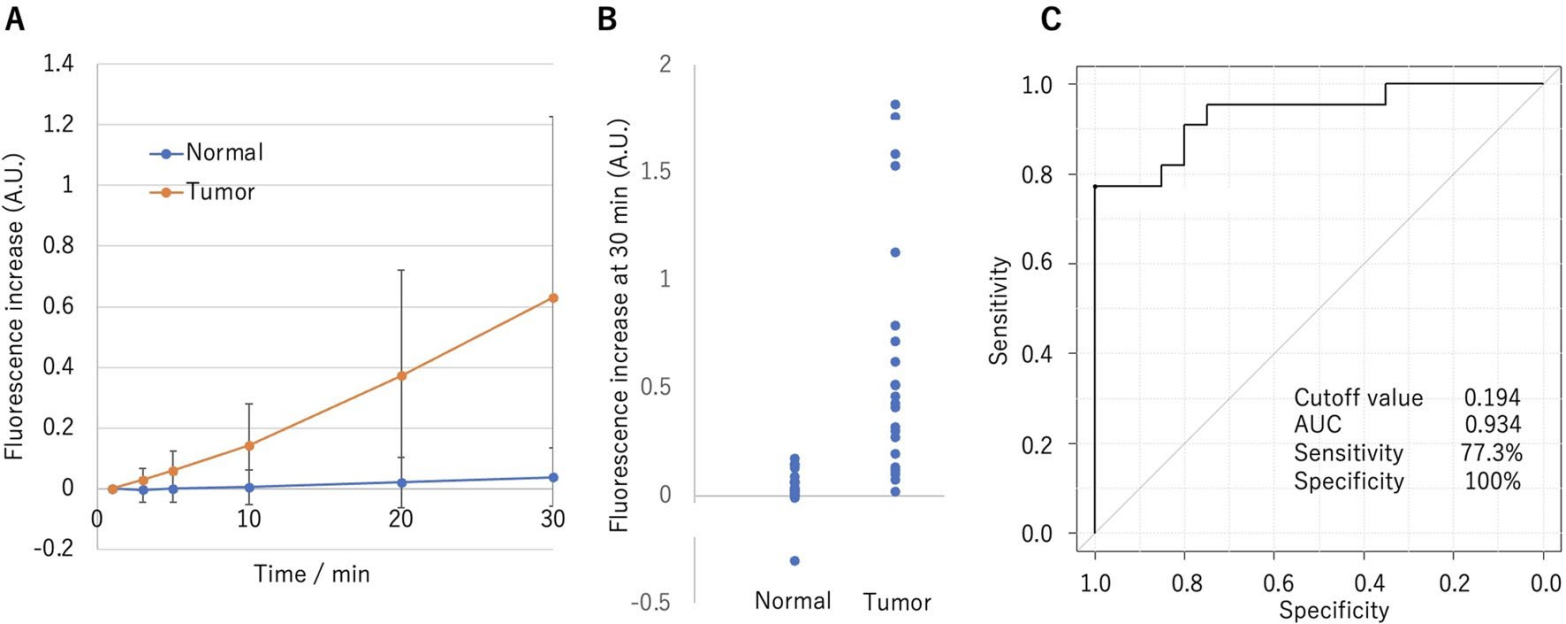
The outcome of ex vivo fluorescence imaging of twenty cases. (**A**) Time-dependent fluorescence increase, calculated as the increase at 30 min from 1 min after addition of gGlu-HMRG. Error bars represent s.d. (**B**) Dot plot of fluorescence increase at 30 min of normal and tumor lesions. (**C**) Receiver operating characteristic curve for all twenty cases. The cutoff value, sensitivity, and specificity of gGlu-HMRG for detection of thymic tumor were calculated for all 20 cases. Sensitivity and specificity were 77.3% and 100%, respectively. AUC, area under curve.

**Figure 3:**
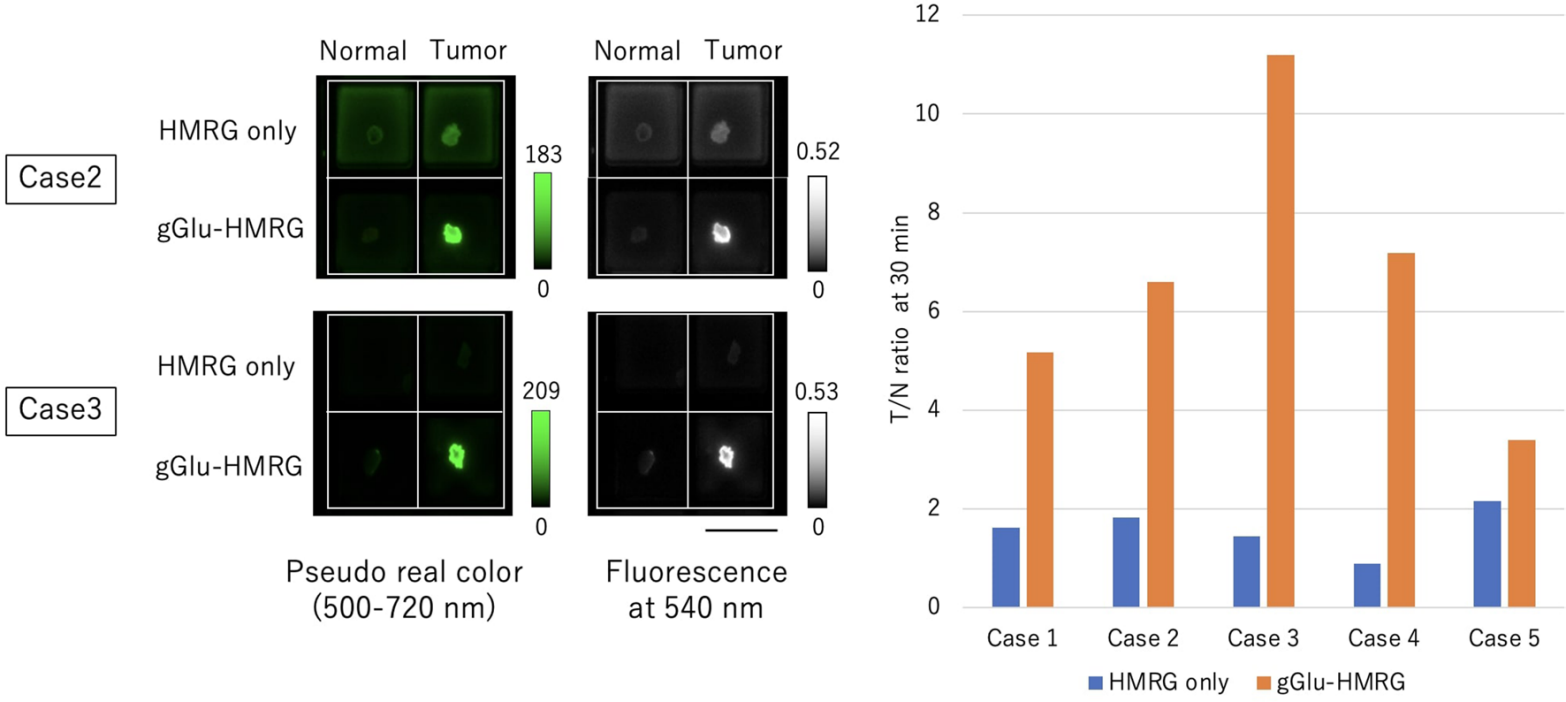
Fluorescence imaging with gGlu-HMRG or HMRG alone. Specimens were incubated with gGlu-HMRG (50 μM) or HMRG (1 μM) for 30 min and the tumor-to-normal (T/N) ratio, i.e., the ratio of fluorescence intensity of the tumor to that of normal tissue at 30 min, was calculated. Five cases were tested, and fluorescence imaging of two representative cases is shown. gGluHMRG showed a higher T/N ratio in cancer tissues, indicating that cancer tissues have higher GGT activity than normal tissues. Scale bar, 10 mm.

We applied 50 μM gGlu-HMRG to surgically resected specimens of normal and tumor tissues from 20 cases (Fig. 1A,B). In 18 of the 20 cases, the tumors fluoresced more strongly than the normal tissues, and gradually became brighter up to 30 min. On the other hand, in two cases, neither the tumors nor the normal cells fluoresced much. The mean fluorescence increases of the tumors and normal tissues in all 20 cases are shown in Fig. 2A, and a dot chart of the fluorescence increase at 30 min is shown in Fig. 2B. In addition, the mean fluorescence increases of specimens from 2013 to 2016 and from 2017 to 2021 are shown separately in Supplementary Fig. S3, and a dot chart of the fluorescence increase of each histological type is shown in Supplementary Fig. S4. From the receiver operating characteristic (ROC) curves, the sensitivity and specificity of this probe were calculated to be 77.3% and 100%, respectively, and the positive predictive value (PPV), negative predictive value (NPV), and accuracy were 100%, 81.5%, and 88.7%, respectively (Fig. 2C). In this experiment, the median tumor-to-normal (T/N) ratio at 30 min in all 20 cases was 7.56 (range: 1.17–1352.82). In an experiment comparing HMRG only and gGlu-HMRG, T/N ratio at 30 min in all five cases examined was higher for gGlu-HMRG, indicating that the fluorescence increase is associated with a specific enzymatic reaction (Fig. 3). When only HMRG was applied, the median T/N ratio was 1.62 (range: 0.89–2.15), which is considered to reflect the difference of color between tumor and normal tissues. When gGlu-HMRG was applied, the median T/N ratio was 6.60 (range: 3.39–11.19), indicating the involvement of GGT.

### Histological and immunohistochemical staining of GGT in tumor samples

**Figure 4:**
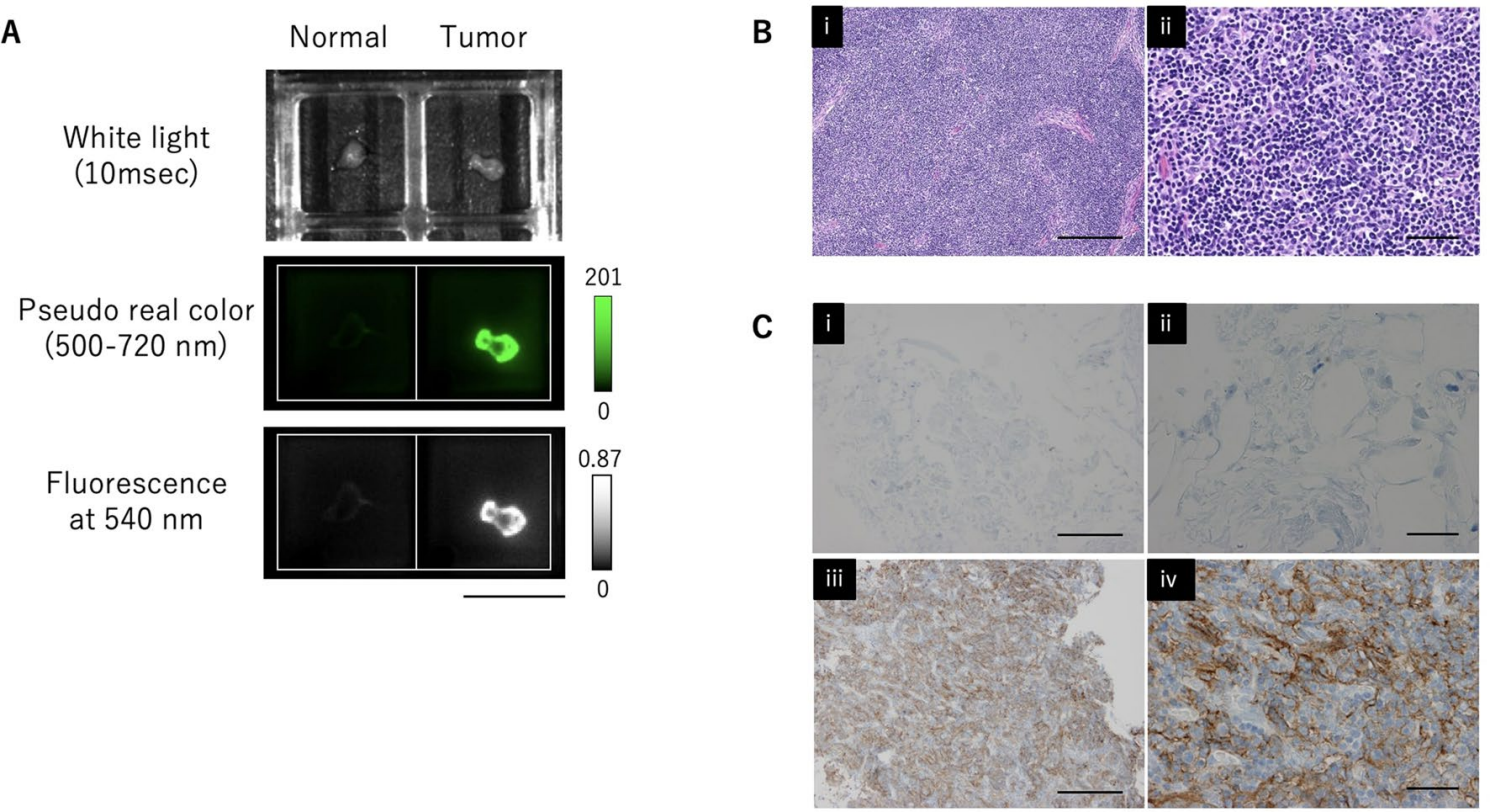
Histological and immunohistochemical staining of GGT in tumor samples. (**A**) Ex vivo fluorescence imaging of pieces of normal and tumor tissue a few millimeters in size after 30 min incubation with 50 μM gGlu-HMRG. Scale bar, 10 mm. (**B**) Histology of thymoma (HE). (i) Scale bar, 250 μm. (ii) Scale bar, 50 μm. (**C**) GGT IHC. Negative in fat tissue (i, ii). High GGT expression in thymoma (iii, iv). (i, iii) Scale bar, 250 μm. (ii, iv) Scale bar, 50 μm.

Hematoxylin and eosin (HE) staining and GGT immunohistochemistry (IHC) were performed on thymoma (type AB) specimens that showed high fluorescence intensity with gGlu-HMRG (Fig. 4A,B). IHC revealed high GGT expression in thymoma while GGT expression was not detected in normal thymic parenchyma and fat tissue (Fig. 4C).

### Application of gGlu-HMRG for specific demarcation of thymoma

**Figure 5:**
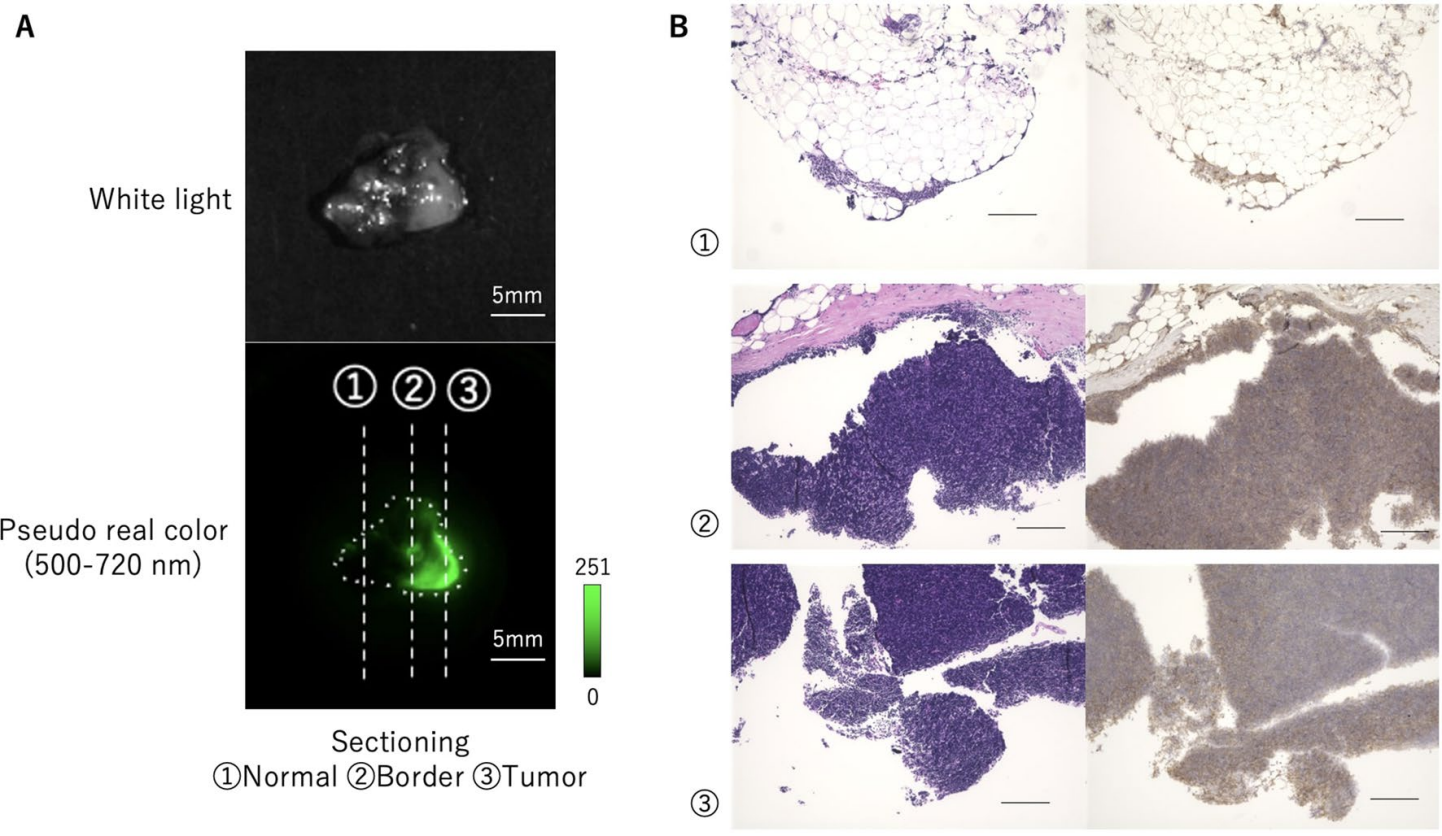
Application of gGlu-HMRG for specific demarcation of thymoma. (**A**) Fluorescence image of surgically resected thymoma specimen containing both normal and tumor tissues after administration of gGlu-HMRG. Tumor area fluoresced well (3) and normal area did not (1), so the border between tumor and normal area could be confirmed (2) by the fluorescence imaging. Scale bar, 5 mm. (**B**) Left: Pathological HE-staining of the same specimen shown in (**A**), sectioned along the indicated lines. Areas of increased fluorescence coincided well with pathologically confirmed thymic tumor regions. Right: IHC staining for GGT. Scale bar, 200 μm.

We next examined whether gGlu-HMRG can visualize the boundaries of tumor lesions in a relatively large surgically resected thymoma specimen. Fluorescence activation was observed at specific regions of the specimen (Fig. 5A). HE-staining confirmed that an area with high fluorescence intensity coincided with thymic tumor region, while non-fluorescent region was normal tissue. We also evaluated the expression of GGT by IHC and confirmed that the tumor showed higher GGT expression than normal tissue (Fig. 5B).

### Fluorescence imaging of specimens treated simultaneously with gGlu-HMRG and a GGT inhibitor

**Figure 6:**
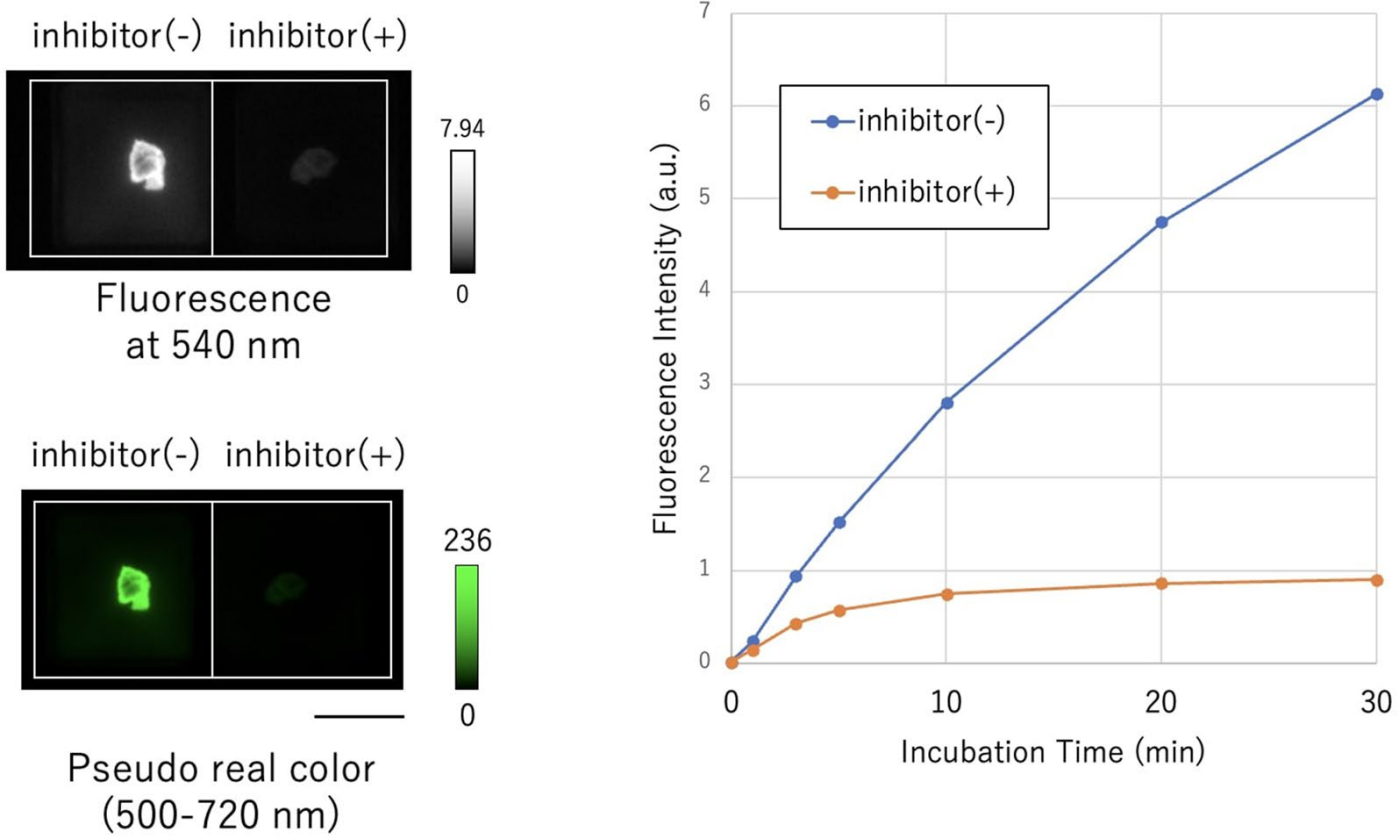
Fluorescence intensity of thymoma tissue in the presence and absence of GGT inhibitor. Fluorescence intensity increased time-dependently in the absence of inhibitor, but the increase was inhibited in the presence of inhibitor. Scale bar, 5 mm. [fluorescent probe] = 50 μM, [GGsTop] = 50 μM.

To confirm that the gGlu-HMRG-derived fluorescence signal is dependent on GGT activity, we performed fluorescence imaging of tissues from one case after application of gGlu-HMRG in the absence or presence of GGsTop, a specific and irreversible GGT inhibitor. The fluorescence increase at 30 min after administration of gGlu-HMRG together with GGsTop was significantly suppressed compared to gGlu-HMRG alone. This result supports the idea that GGT activity is required to cleave gGlu-HMRG in thymoma, and that GGT activity is higher in the cancer tissue than in normal tissue (Fig. 6).

### Evaluation of gene expression in nucleic acids extracted from thymoma tissue

Nucleic acids were extracted from thymomas and normal tissues, and the expression level of GGT1 was evaluated by quantitative polymerase chain reaction (qPCR) (Supplementary Fig. S5). This experiment was performed to measure the difference in GGT1 expression between cases that fluoresced and did not fluoresce by ex vivo fluorescence imaging. In the cases that fluoresced, GGT1 was more highly expressed in tumor tissues than in normal tissues (Cases 1–3). On the other hand, in the case that did not fluoresce, GGT1 was rarely observed in either normal or tumor tissue (Case 4). Among several GGT subtypes, we focused on GGT1 because this subtype is reported to be involved in both glutathione catabolism and anticancer drug resistance.

## Discussion

The efficacy of surgical resection for stage I-II thymic epithelial tumors was established in the 1980s^8,9^. Large-scale evaluations in Japan and by International Thymic Malignancy Interest Group (ITMIG) confirmed that the results are excellent (thymoma: 5-year overall survival rate of approximately 95%, 10-year survival rate of approximately 88%; thymic carcinoma: 5-year overall survival rate of approximately 85%, 5-year recurrence-free survival rate of approximately 80%), and surgical resection by median sternotomy became the standard treatment for patients with stage I–II thymic epithelial tumors^10–13^. Currently, however, thoracoscopic surgery and robotic surgery are considered preferable to median sternotomy, being associated with lower blood loss, shorter admission time, and shorter chest tube time^14^. However, for these procedures, it is essential to visualize the tumor tissues accurately. This is because thymic carcinoma and invasive thymoma tend to invade the surrounding organs, and it is sometimes difficult to determine how far the tumor has invaded. In such cases, this fluorogenic probe could be useful, for example, by topically spraying it on the resected surface of the remaining thymus gland or other organs when residual tumor is suspected, or by applying it to cross sections of the resected specimen to assess the surgical margins. Although we used the bright green-emitting probe for topical application based on its clear visibility, the fluorophore could be replaced with a different fluorescent scaffold depending on the detection filters or desired tissue penetration depth of light. For example, we have developed several red to near-infrared (NIR) probes targeting GGT based on the spirocyclization design strategy. These probes can also be applied for the visualization of thymomas and thymic carcinomas^15,16^.

Previous reports from our laboratory have shown that the sensitivity/specificity of gGlu-HMRG for detection of lung cancer, hepatocellular carcinoma, and breast cancer are 43.8/84.9%, 48/96%, and 92/94%, respectively^4–6^. Here, we obtained sensitivity and specificity values of 77.3% and 100%, respectively, in fluorescence imaging of thymoma surgical specimens with gGlu-HMRG. One reason for the relatively high sensitivity for thymic epithelial tumors, as well as for breast cancer, may be that the normal tissue has a large fatty component, in which gGlu-HMRG would not be activated. These results suggest that gGlu-HMRG should be useful for the detection of thymic epithelial tumors, as well as breast cancers. In addition, although the fluorescence intensity at 30 min was measured in this paper, differences in fluorescence intensity between tumor and normal tissues were clearly observable within just a few minutes after the application of gGlu-HMRG (Supplementary Fig. S6). The ability to assess the surgical margin in the operating room would allow faster and more convenient assessment than conventional pathological evaluation.

We also confirmed that the boundaries between tumor and normal tissues in serial specimens could be clearly distinguished by gGlu-HMRG, even when it was difficult to see with the naked eye. Although some normal thymic tissues and vascular endothelium were weakly stained, tumor tissues were strongly stained in the large majority of cases. In addition, GGsTop suppressed the fluorescence increase. These findings indicate that GGT activity is high in the tumor environment and the fluorescence increase of gGlu-HMRG is caused by the enzymatic activity of GGT.

A recent survey conducted by Japan Association for Research on the Thymus (JART) showed a good prognosis for patients who underwent complete gross resection of stage IVa thymoma with dissemination; the 10-year survival rate was 88.6%^17^. Furthermore, if complete gross resection is not possible, "debulking" surgery contributes to an improved prognosis^18^, so there is a need to identify small nodules intraoperatively, especially to find pleural seeding nodules that cannot be seen with the naked eye. Thus, rapid fluorescence imaging of disseminated nodules with gGlu-HMRG, for example, by administration in the thoracic cavity, may be of great help to general thoracic surgeons. Our findings indicate that gGlu-HMRG may be an effective tool for rapid intraoperative imaging of thymic epithelial tumors with high sensitivity and specificity.

## Methods

### Enzyme-activatable fluorescence probe and reagents

gGlu-HMRG, an activatable fluorescence probe targeting GGT, and HMRG were prepared as previously described^7^. They were stored as 10 mM solutions in dimethyl sulfoxide (DMSO, Sigma-Aldrich, St. Louis, Missouri) at − 80 °C. Before ex vivo application to clinical specimens, the DMSO stock solution was thawed at room temperature and diluted to a final concentration of 50 μM (gGlu-HMRG) or 1 μM (HMRG) in phosphate-buffered saline (PBS, Life Technologies, Carlsbad, California). All organic solvents and reagents were commercial products of guaranteed grade and were used without further purification. Water was doubly distilled and deionized by a MilliQ water system before use.

### Clinical samples

22 patients with thymoma or thymic carcinoma who underwent surgery between February 2013 and January 2021 at the University of Tokyo were included in the study. These are relatively rare diseases and only a few cases are operated on each year. In addition, only tumors larger than 2 cm were selected in this study to avoid compromising pathological assessment. Twenty patients were enrolled for the ex vivo fluorescence imaging study (Figs. 1, 2, 3, 4 and Supplementary Table S1), one patient was enrolled for the experiment involving application to serial sections of tumor and normal tissue (Fig. 5) and one patient was enrolled for the chemical inhibition experiment (Fig. 6). Some patients were included in plural analyses. Written informed consent was obtained from all patients, and this study was approved by the ethics committee of The University of Tokyo and the local ethics committees. All experiments were performed in accordance with guidelines and regulations approved by the ethics committees. All specimens were taken intraoperatively. Fluorescence images, except for the inhibition experiment and the experiment comparing HMRG only and gGlu-HMRG, were collected within a day after resection. For the inhibition experiment, frozen specimens were thawed at room temperature and used within 6 months. With regard to the experiment comparing HMRG only and gGlu-HMRG, data from frozen and raw specimens are mixed.

### Ex vivo fluorescence imaging study of patients’ specimens

Images were captured with a Maestro in vivo imaging system (PerkinElmer) before and at 1, 3, 5, 10, 20, and 30 min after applying 50 μM gGlu-HMRG solution in PBS containing 0.5% v/v DMSO to tumor and normal specimens at room temperature. A sufficient amount of solution was used to ensure that the specimen was fully immersed. The excitation and emission wavelengths were 445–490 nm and 515 nm long pass, respectively. The Maestro’s tunable filter was automatically switched in 10 nm increments from 500 to 720 nm, while the camera sequentially captured images at each wavelength interval. Fluorescence at 540 nm was extracted, and fluorescence intensities were quantified by drawing regions of interest (ROIs) with the Maestro software. Exposure time was set at 50–100 ms depending on the fluorescence intensity. From 2013 to 2016 (8 cases in total), 50 μM of probe was applied to a relatively large specimen (about 1 cm) and three ROIs were set for both tumor and normal tissues, then the mean fluorescence intensity was calculated using Maestro software. From 2017 to 2021 (12 cases in total), specimens were cut into pieces a few millimeters in size, which were placed individually in wells of an 8-well chamber (μ-Slide 8 well; Ibidi), and 200 μl of probe solution was added to each well. ROIs were set for entire specimens. In five of the latter cases HMRG only (1 μM) was applied in order to examine the effect of the difference in color between tumor and non-tumor tissues, as the fluorescence intensity of reddish non-tumor tissues tends to be under-recorded. In one experiment, we performed fluorescence imaging of serial specimens of tumor and normal tissue after topically applying 2 ml of probe to the entire specimen. Increase in fluorescence intensity was calculated by subtracting the initial fluorescence intensity from that measured after incubation for 30 min with the probe. Fluorescence images were also obtained after treating paired samples with gGlu-HMRG (50 μM) with or without GGsTop (50 μM; Wako Pure Chemical Industries), which is a specific irreversible inhibitor of GGT, under the same conditions^19^. This inhibition experiment was performed using frozen specimens, which were allowed to thaw naturally at room temperature.

### Histological analysis

Resected specimens were immediately fixed with formalin for at least 24 h and embedded in paraffin. Paraffin-embedded tissues were sectioned at 4 μm thickness and stained with HE for histopathological evaluation. Certified pathologists made diagnosis according to the 5th edition of the World Health Organization (WHO) classification of thymic tumors^20^. Tumor stage was determined according to the 8th edition of the TNM staging system of the Union for International Cancer Control (UICC) and the Masaoka-Koga classification system^8,21^.

### Immunohistochemical analysis of GGT expression

For IHC, sections were deparaffinized in Histo-Clear, sequentially washed in 100%, 90%, 80%, and 70% ethanol, and then washed in PBS. After heat-induced antigen retrieval (Tris–EDTA buffer, pH 9) using a microwave oven, each slide was pre-incubated in 3% H_2_O_2_ for 20 min, reacted with primary antibodies (mouse polyclonal antibody, H00002678-M01; Abnova, Taipei, Taiwan) in 5% skim milk for 90 min, and reacted with secondary antibodies (Takara POD conjugate anti Mouse for GGT, Japan) for 30 min at room temperature. Each slide was visualized with a 3,3′-diaminobenzidine tetrahydrochloride (DAB) detection kit (Product number: MK210, TaKaRa), and counter-stained with hematoxylin. GGT antibody was diluted to 1/3000 and the DAB reaction time was 5 min.

### qRT-PCR analysis

Frozen specimens of thymomas and normal tissues were slightly shredded with scissors and total mRNA was extracted using TRIzol RNA Isolation Reagent (Gibco, #10296028). Expression level of GGT1 was analyzed by one-step probe qPCR using Luna Universal Probe One-Step RT-qPCR Kit (NEB, #E3006) and IDT PrimeTime^®^ qPCR Assays composed of Forward/Reverse primer pair and PrimeTime Probe. Primers were hGGT1 for GGT1 expression, and hGAPDH and hHPRT1 as internal standards. Primer and probe information is described in Supplementary Table S2. The mixture information is shown in Supplementary Table S3. The samples were centrifuged and each sample was measured by triplicate using a LightCycler^®^480 System II (F. Hoffmann-La Roche Ltd, Basel, Switzerland). The mRNA expression levels of GGT1 were expressed relative to internal standards and compared between tumor and normal.

### Statistical analysis

Statistical analyses were carried out using software R 3.3.2 (R Foundation for Statistical Computing, Vienna, Austria). Sensitivity, specificity, PPV, NPV, and accuracy were evaluated from the ROC curves.

## Supplementary Information

Supplementary Information.

## Data Availability

All data and materials are available upon request. Correspondence and requests for materials should be addressed to Y.U. (email: uranokun@m.u-tokyo.ac.jp) or J.N. (email: nakajima-tho@h.u-tokyo.ac.jp).
