# Supplementary material for: Rapid imaging of thymoma and thymic carcinoma with a fluorogenic probe targeting γ-glutamyltranspeptidase
Source: Sci Rep. 2023 Mar 7;13:3757. doi: 10.1038/s41598-023-30753-2 (PMC9992351; doi:10.1038/s41598-023-30753-2)
Supplement: Supplementary file 1 — Supplementary Information. [file 41598_2023_30753_MOESM1_ESM.pdf]

Supporting Information

Rapid Imaging of Thymoma and Thymic Carcinoma with a Fluorogenic Probe Targeting γ-Glutamyltranspeptidase

Daisuke Yoshida[1,2], Mako Kamiya[2], Shun Kawashima[1,2], Takafusa Yoshioka[1,2], Haruaki Hino[2,3], Atsuki Abe[2], Kyohhei Fujita[2], Ryosuke Kojima[2], Aya Shinozaki-Ushiku[4], Yasuteru Urano[2,5*] and Jun Nakajima[1*]

[1]Department of Thoracic Surgery, Graduate School of Medicine, The University of Tokyo, 7-3-1 Hongo, Bunkyo-ku, Tokyo 113-8655, Japan. [2]Laboratory of Chemical Biology and Molecular Imaging, Graduate School of Medicine, The University of Tokyo, 7-3-1 Hongo, Bunkyo-ku, Tokyo 113-0033, Japan. [3]Department of Thoracic Surgery, Kansai Medical University, 2-3-1 Shinmachi, Hirakata City, Osaka 573-1191, Japan. [4]Department of Pathology, Graduate School of Medicine, The University of Tokyo, 7-3-1 Hongo, Bunkyo-ku, Tokyo 113-8655, Japan. [5]Graduate School of Pharmaceutical Sciences, The University of Tokyo, 7-3-1 Hongo, Bunkyo-ku, Tokyo 113-0033, Japan.

*To whom correspondence should be addressed.
E-mail: uranokun@m.u-tokyo.ac.jp /nakajima-tho@h.u-tokyo.ac.jp

Supplemental Figures.

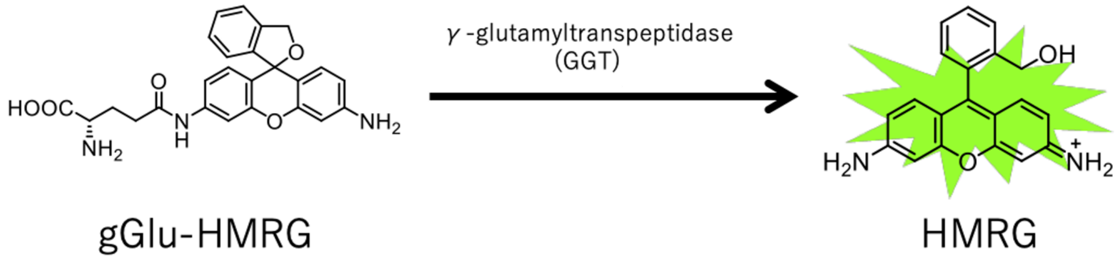

Supplementary Figure S1. Reaction scheme of gGlu-HMRG, which is non-fluorescent, but was converted to a highly fluorescent HMRG uopn reaction with γ-glutamyltranspeptidase.

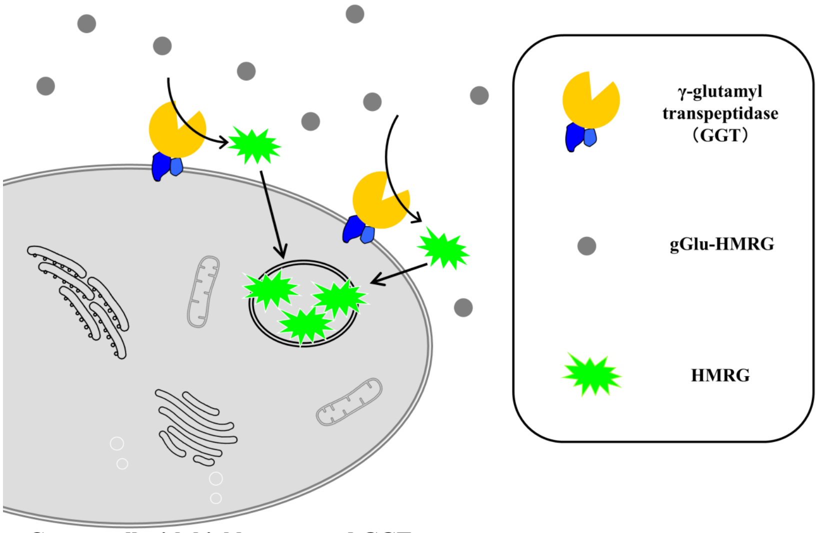

Supplementary Figure S2. Activation mechanism of gGlu-HMRG by γ-glutamyltranspeptidase at cancer-cell surface, followed by the accumulation of the produced HMRG into lysosome.

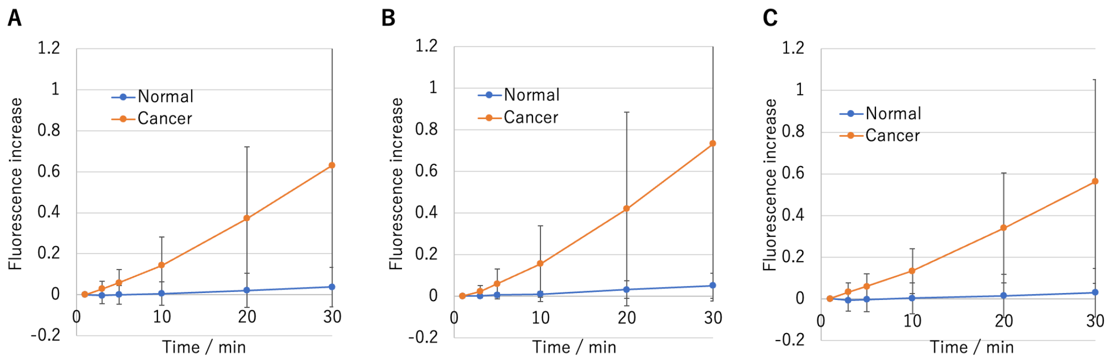

Supplementary Figure S3. The mean fluorescence increases of the tumors and normal tissues. (A) The mean fluorescence increases of whole periods. Median T/N ratio at 30 min is 7.56. (B) The mean fluorescence increases from 2013 to 2016. Median T/N ratio at 30 min is 7.56. (C) The mean fluorescence increases from 2017 to 2021. Median T/N ratio at 30 min is 9.92. Error bars represent s.d.

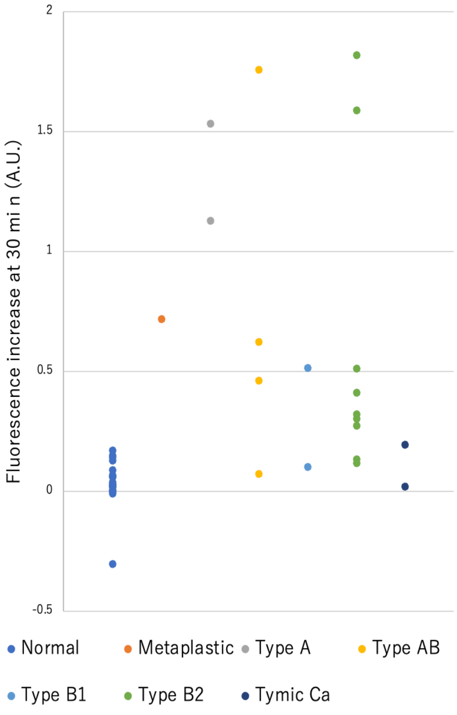

Supplementary Figure S4. Dot chart of the fluorescence increase at 30 min of each histological type. In these 20 cases, we found no relationship between fluorescence intensity and histological types.

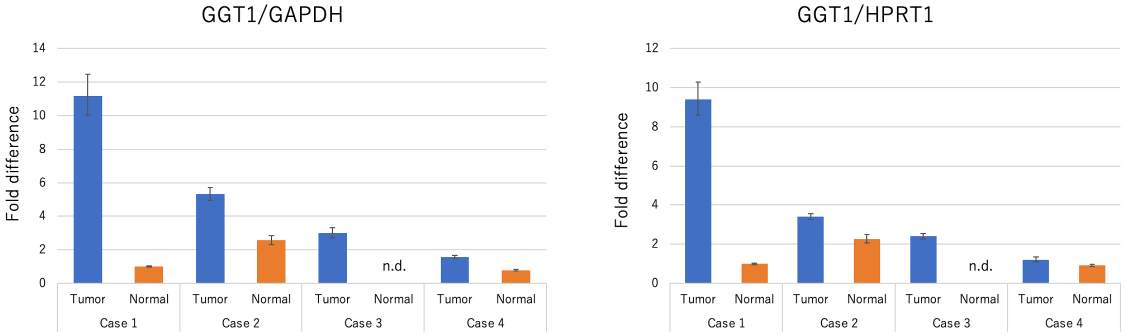

Supplementary Figure S5. Quantitative PCR analysis of GGT1 expression in thymoma tissues. In the cases that fluoresced, GGT1 was more highly expressed in the tumor than in normal lung tissue (Case 1-3). On the other hand, in the case that did not fluoresce, GGT1 was rarely observed in both normal and tumor tissues (Case 4). Fold difference is against Normal in Case 1 (calculated by 2^ΔΔCt value). Error bars represent standard deviations (n=3, technical replicates).

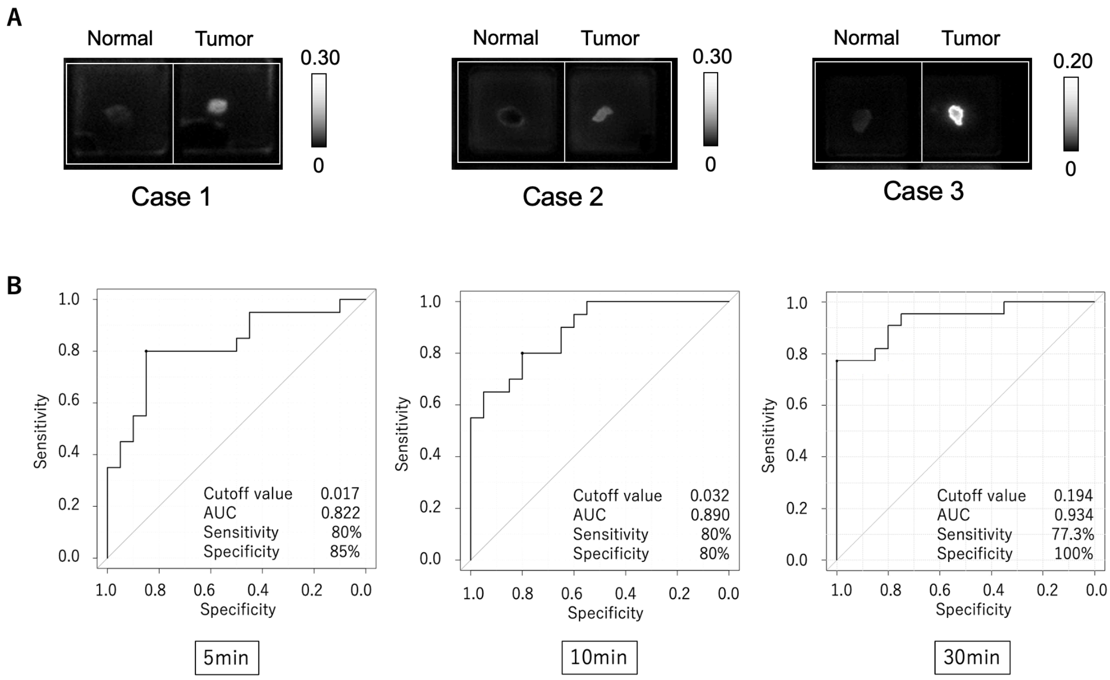

Supplementary Figure S6. Rapid imaging within a few minutes after the application of gGlu-HMRG. (A) Fluorescence images of three representative cases at 540 nm 5 minutes after the application of gGlu-HMRG. Differences in fluorescence intensity between tumor and normal tissues were clearly observable. (B) ROC curves at 5, 10, and 30 minutes. Over time, the AUC increases, but it already has a sufficiently high AUC at 5 minutes.

Characteristics			
Patients' age, yr, mean (range)			60.09 (34-78)
Gender	Male/Female		11/11
Tumor size, mm, mean (range)			52.95 (20-105)
Histology	Thymoma		20
		TypeA	2
		TypeAB	5
		TypeB1	2
		TypeB2	10
		TypeB3	0
		Metaplastic	1
	Tymic carcinoma		2
Masaoka-Koga classification	I		5
	IIa		8
	IIb		3
	III		6
	IV		0
TNM classificasion	I		17
	II		1
	IIIa		3
	IIIb		0
	IVa		1
	IVb		0

Supplementary Table S1. Characteristics of 22 surgically resected thymic tumors used for *ex vivo* fluorescence imaging with gGlu-HMRG.

GGT1	Assay name: Hs.PT.58.22838435<br>Probe: 5'-/ 56-FAM/TGGACAGTT /ZEN/CAGTGA TTTGCCTGAGG /31ABkFQ/-3'<br>Primer 1: 5'-TCTGAGGAAGAGGTGCTCTC-3'<br>Primer 2: 5'-CCTTCTCGTTCTCCTGCTG-3'
GAPDH	Assay name: Hs.PT.39a.22214836<br>Probe: 5'-/ 56-FAM/ AAGGTCGGA/ZEN/GTCAACGGA TTTGGTC/31ABkFQ/-3'<br>Primer 1: 5'-ACATCGCTCAGACACCATG-3'<br>Primer 2: 5'-TGTAGTTGAGGTCAATGAAGGG-3'
HPRT1	Assay name: Hs.PT.58v.45621572<br>Probe: 5'-/ 56-FAM/ AGCCTAAGA/ZEN/TGAGAGTTCAAGTTTGG /31ABkFQ/-3'<br>Primer 1: 5'-TTGTTGTAGGATATGCCCTTGA-3'<br>Primer 2: 5'-GCGATGTCAATAGGACTCCAG-3'

Supplementary Table S2. Information of IDT PrimeTime® Probe used in this study.

	GAPDH	HPRT1	GGT1
Luna Universal Probe One-step Reaction Mix (NEB)	10 μL	10 μL	10 μL
Luna Warm Start® RT Enzyme Mix (NEB)	1 μL	1 μL	1 μL
IDT PrimeTime® qPCR Assay solution*	1 μL	0.2 μL	1 μL
Sterilized water (Rnase-free)	7.5 μL	8.3 μL	7.5 μL
RNA extracted from tissue (~200 ng) **	≈0.5 μL	≈0.5 μL	≈0.5 μL
total	≈20 μL	≈20 μL	≈20 μL

Supplementary Table S3. Quantitative PCR reagent preparation information.

* Mixture of Forward/Reverse primers and PrimeTime Probe. For every gene, the final concentration of Forward/Reverse primers and PrimeTime Probe was adjusted to 500 μM and 250 μM respectively.

** To adjust the amount of RNA to 200 ng in all samples, the volume of solution was varied depending on the concentration of RNA in the solution. The RNA concentration ranged from 276.7 ng/μL to 445.8 ng/μL and the volume ranged from 0.45 μL to 0.72 μL.
